# School-Based Pre- and Post-Intervention Tests Assessing Knowledge about Healthy Lifestyles: A National School Health Awareness Campaign on Children Aged between 3 and 12 Years Old

**DOI:** 10.3390/children11020213

**Published:** 2024-02-07

**Authors:** Charbel Moussi, Léa Tahan, Peter Habchy, Ogarite Kattan, Alain Njeim, Leila Abou Habib, Wassim El Bitar, Béchara El Asmar, Mirna N. Chahine

**Affiliations:** 1 Faculty of Medical Sciences, Lebanese University, Hadath P.O. Box 3, Lebanon; c.moussa@st.ul.edu.lb (C.M.); lea.tahan@st.ul.edu.lb (L.T.); p.habchy@st.ul.edu.lb (P.H.); o.kattan@st.ul.edu.lb (O.K.); a.njeim@st.ul.edu.lb (A.N.); 2 Lebanese Association of the Knights of Malta (Order of Malta Lebanon), Vanlian Bldg, 6th Fl. City Rama Str. Dekwaneh, Beirut P.O. Box 11-4286, Lebanon; leila.sebeel@yahoo.com (L.A.H.); wassim.elbitar@chirec.be (W.E.B.); bechara.elasmar@hdf.usj.edu.lb (B.E.A.); 3 Department of Pediatrics, Bellevue Medical Center University Hospital, Mansourieh P.O Box 295, Lebanon; 4 Faculty of Medicine, Saint Joseph University, Beirut P.O. Box 17-5208, Lebanon; 5 Department of Cardiology, Hotel-Dieu de France Hospital, Achrafieh, Beirut P.O. Box 11-5190, Lebanon; 6 Basic Sciences Department, Faculty of Medical Sciences, Lebanese University, Hadath P.O. Box 3, Lebanon; 7 Foundation-Medical Research Institutes (F-MRI^®^), Achrafieh, Beirut P.O. Box 64, Lebanon; 8 Foundation-Medical Research Institutes (F-MRI^®^), 1211 Geneva, Switzerland

**Keywords:** children, Lebanon, awareness campaign, schools, intervention, health, knowledge and practice

## Abstract

In response to the diverse health challenges faced by today’s youth and their extensive time spent in schools, we conducted a school health awareness campaign aimed at prioritizing well-being and academic performance. This analytical longitudinal study, spanning 27 schools in Lebanon, aimed to assess the impact of the awareness campaign on the health knowledge and practices of 5-, 8-, and 11-year-old students throughout pre- and post-intervention testing focused on general health and healthy habits, employing 11, 14, and 15 questions tailored to 5, 8, and 11 year olds, respectively. The questions covered various aspects, including sleep, personal and dental hygiene, nutrition, physical activity, addiction, security measures, and bullying. Out of the 7100 students who participated, 16.11% (1144 students) were evaluated before and after the campaign. The results indicated a significant increase in health awareness post-intervention across all age groups. For instance, in 5-year-olds, limited awareness decreased from 91.6% to 36.38%, while adequate awareness rose from 8.03% to 62.3%. Improvement varied across health knowledge topics, with security-related questions showing the highest enhancement. Factors such as governorate, normal-weight status, and close supervision influenced improvement. However, no significant correlations were found with school type, size, gender, age, nationality, parental factors, or comorbidities. The study concludes that the school health awareness campaign successfully heightened children’s health awareness, advocating for the integration of regular health promotions into the standard educational curriculum.

## 1. Introduction

### 1.1. General Background

Health promotion empowers individuals to take control of the factors that influence their health [1]. Providing young children with health promotion initiatives such as proper nutrition, regular exercise, good oral hygiene, screen time, and sufficient sleep is an investment in their future. It can improve their physical health, mental health, overall well-being, quality of education, and ultimately, their quality of life. It is crucial for individuals to acquire this knowledge during the most important developmental stage of life, which is childhood [2,3,4].

Due to the length of time that young children spend in schools, it is essential for educators to promote healthy lifestyles and teach preventative measures [5]. This is validated by the review of previously implemented health initiatives in schools, which showed that these institutions were recognized for their critical role in addressing social and health-related issues due to their strategic capacity to interact with children and families [6].

For instance, the GiochiAMO project in Italy represents a noteworthy initiative aiming to increase primary school children’s knowledge of the food pyramid, adherence to the Mediterranean diet, and promotion of physical activity through engaging games. This innovative project served as a model for creating school environments that foster a healthy lifestyle [7].

In addition, Sacchetti et al., (2015) conducted a three-year school-based health intervention, targeting physical activity and dietary habits in primary school pupils. The study revealed reduced excess weight, improved dietary practices, and enhanced fruit and vegetable intake [8].

The leading causes of death in adults, such as heart disease, cancer, stroke, pneumonia, diabetes, suicide, and HIV, are well-known. McGinnis and his colleagues conducted an analysis and determined that the most significant risk factors contributing to these fatal diseases over the long term are tobacco use, diet, alcohol consumption, infections, sexual behavior, and drug use [9]. Researchers highlighted that many of the latter underlying risk factors are behavioral and related to lifestyle choices that can be significantly impacted by education in schools [10]. In addition, health education and practices can effectively prevent the outbreak and transmission of communicable diseases [11,12]. Furthermore, according to Michael et al., the incorporation of health-related elements, such as health education and health services, can influence educational achievements [13].

To sum it up, prioritizing early childhood health is crucial for preventing unfavorable outcomes in the short and long run.

### 1.2. Specific Background

On an international scale, the School Health and Nutrition (SHN) program held in Nepal is an illustration of such initiatives: the program was successful in enhancing the physical, emotional, and mental health of students, thereby achieving its objective. Despite facing some challenges, all parties involved in the program acknowledged its positive impact on the students, schools, and communities [11,14]. An additional project conducted in Lithuania demonstrated that an eight-month physical education program had favorable outcomes on the physical activity and emotional well-being of primary school children [6].

In another study conducted in East Hungary, it was shown that an intervention using interactive techniques and digital resources successfully improved students’ knowledge and health behavior [15].

Moving to a national scale, despite the ongoing economic and health crises that have affected Lebanon, the Ministry of Public Health (MOPH) did not fail to implement and improve various school health programs to promote the health and well-being of young people, including health education integrated into the public-school curriculum since 1987 [12]. In addition, the Global School-based Student Health Survey (GSHS) developed by the WHO provided the Lebanese Ministry of Education and Higher Education (MEHE) with valuable data to enhance students’ health in a healthy school environment. A total of 5708 students in grades 7–12 from 56 public and private schools answered a self-administered 88-item questionnaire related to the use of alcohol, eating habits, drug usage, hygiene, mental health, physical activity, protective factors, sexual behavior, and cigarette use. The results highlighted differences in health outcomes by grade level, sex, and school type [12].

Another national project conducted by the Modern University for Business and Science (MUBS) under the name of “Supporting School Health in Disadvantaged Rural Areas in Bekaa and Southern Lebanon”, aimed to pilot the school health project set by the WHO in five public schools in South and Bekaa, Lebanon. Medical examination and health education were the main focuses of this research. A questionnaire was filled out by both the students and the parents at the baseline and after the intervention/workshops. The results found that the parent workshops increased their knowledge regarding students’ health and stronger bonds were built with their children [16]. However, similar projects on a wider geographical scale are needed.

The Lebanese health curriculum has many weaknesses and a high percentage of schools have inadequate school health quality [14,17]. In addition, Akel et al.’s findings served as a valuable assessment of current practices and a starting point for encouraging future improvement efforts [18].

### 1.3. Gap in the Knowledge

Globally, young children’s health faces unique challenges amidst evolving societal norms and rapid lifestyle changes. This is particularly pronounced in Lebanon, a country navigating economic and health crises, where the significance of prioritizing early childhood health is accentuated. Despite commendable efforts by the Ministry of Public Health and initiatives like the Global School-based Student Health Survey [12], notable gaps persist, notably in the quality of the health curriculum and the overall school health environment. Since society undergoes constant and rapid change, and intergenerational differences are both normal and essential, it is crucial that schools provide ongoing and enhanced health programs for children.

### 1.4. Objectives

In this project held by the Order of Malta Lebanon, we determined the impact of a school health campaign on the health status of children aged between 3 and 12 years in Lebanon by performing an awareness campaign for all school students aged between 3 and 12 years preceded and followed by short pre- and post-interventional tests, respectively, on students aged 5, 8, and 11 years old. We then identified the factors associated with the improvement of health Knowledge and Practice following the intervention.

## 2. Materials and Methods

Our study aimed to investigate the impact of a school-based health awareness campaign on children aged 5, 8, and 11 years old in Lebanon.

### 2.1. Study Design and Population

**Figure 1:**
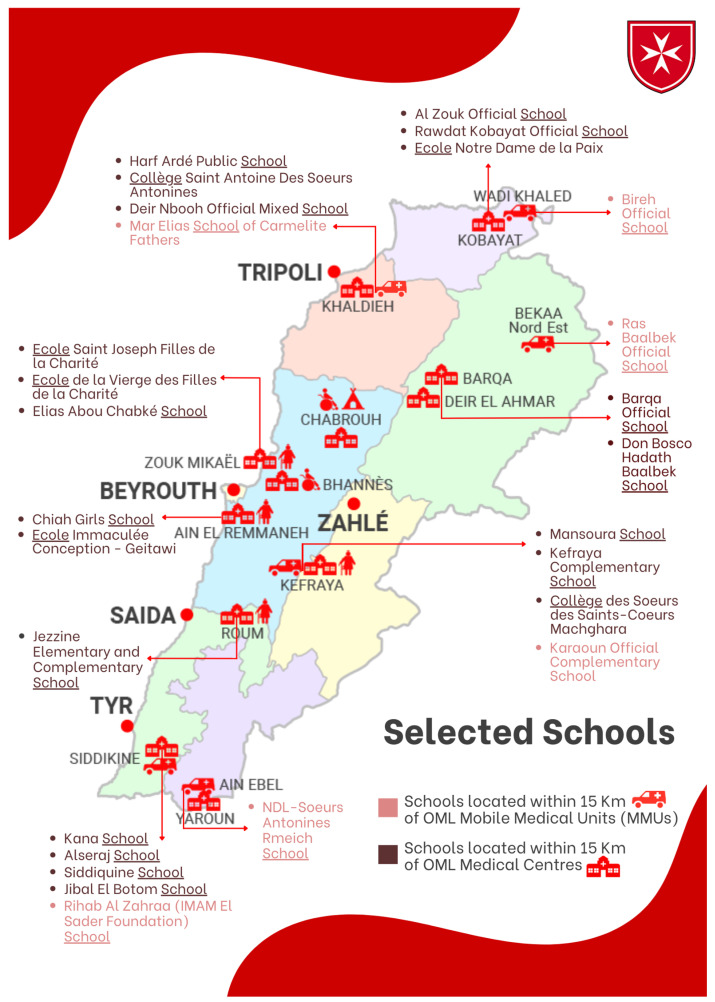
Selected schools in proximity of OML centers and MMUs. OML: Order of Malta Lebanon; MMUs: Mobile Medical Units.

An analytical longitudinal study was carried out in 27 schools widely distributed all over Lebanon, and in proximity to OML medical centers and MMUs (Medical Mobile Units). In total, 16 public schools and 11 semi-private ones with variable sizes were recruited in our project (Figure 1). We selected children of any nationality between 3 and 12 years of age (from kindergarten to grade 7) who are in schools located at a perimeter maximum of 15 km surrounding the OML medical centers and Mobile Medical Units (MMUs). For each center or MMU, around 2 to 3 public schools and 1 semi-private school were chosen. Excluded from the study were students in the mentioned grades who fell outside of the specified age range.

**Figure 2:**
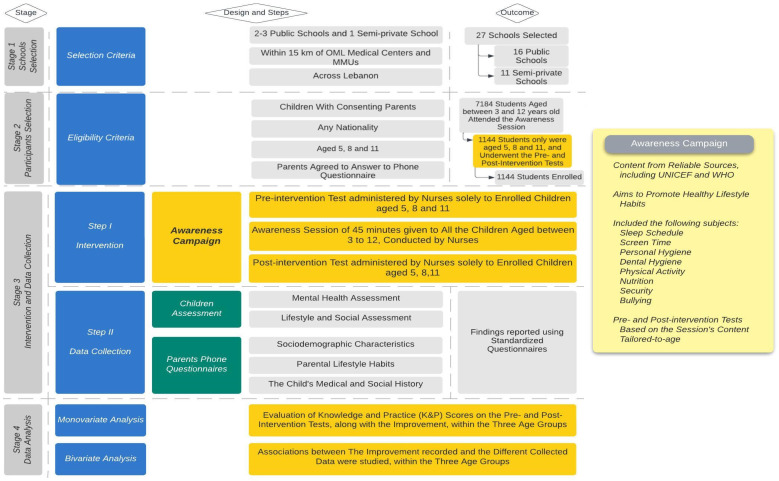
Study flow. OML: Order of Malta; MMUs: Mobile Medical Units; highlighted in yellow are the key elements directly related to the study objectives, serving as the main workflow of the research.

Two important steps were conducted for the aim of this project (Figure 2): 

In the first step of this study, nurses carried out an awareness campaign of 40 to 45 min about lifestyle habits (sleep, screen, personal hygiene, physical activity, nutrition, bullying) through PowerPoint presentations and live demonstrations (whiteboard, drawings…) for all school students aged between 3 and 12 years. Prior to the intervention, nurses also administered a brief pre-test, and subsequently, a post-test solely for students aged 5, 8, and 11 years old. This process took place between 14 and 19 November 2022.

In the second step, OML representatives (medical staff) performed phone interviews with children’s parents using a questionnaire about any physical or mental condition the students may be experiencing. In addition, socio-demographic characteristics were assessed. The phone call took 30 to 35 min per parent. It was carried out within the first ten days of December 2022.

Lebanese university medical students were recruited as OML medical staff to interview children’s parents and complete the medical file of each child. They were trained to verify the parents’ consent, to deliver a message of introduction provided by the OML communication department, and to ensure the good quality of their performance.

### 2.2. Tools and Data Collection

The OML nurse prepared the PowerPoint presentations and pre- and post-intervention tests, which were then reviewed and edited by the research department. These pre- and post-intervention tests transformed into questionnaires by the authors, comprised identical questions, that were customized for 5-, 8-, and 11-year-old students, incorporating 11, 14, and 15 questions, respectively. Each questionnaire included sections covering OML center-related inquiries, student profiles, and diverse topics such as sleep, personal and dental hygiene, nutrition, physical activity, addictions, security, bullying, and the overall knowledge and habits of the students in these domains. The questions in the test were designed based on the content of the PowerPoint presentations, with the language and terminology adapted to suit the age of the children (5, 8, and 11 years old). The PowerPoint presentations were sourced from various online platforms recommended by UNICEF [19,20,21], WHO [22], and other sources [23,24]. In addition to the presentations, live demonstrations were conducted using whiteboards and drawings. Before and following the intervention, face-to-face interviews for 4 to 5 min were conducted, and the responses were recorded on Google forms using the pre- and post-intervention tests that were also prepared. All questionnaires were translated from English to French and Arabic languages using the inverted method of Fortin [25]. The authors first translated it from English to French/Arabic. Then, the Arabic/French versions were translated into English by a healthcare professional/translator to compare the agreement of the instrument. A preliminary test was carried out with ten persons who were not part of the sample to validate the understanding and clarity of the questionnaire items. At the end of the preliminary test, the questionnaire was modified as necessary [25].

### 2.3. Variables

– Sociodemographic and other students’ characteristics:

Sociodemographic factors, comorbidities, habits, BMI, mental health of children as well as the occupation, income, health knowledge, level of education, marital status, smoking status, and comorbidities of parents.

As part of our campaign (but not part of this article), all of these factors were collected through a physical exam of children by both nurses and medical doctors. Additionally, nurses conducted face-to-face interviews with the children to gather more information about their mental health and lifestyle habits. Moreover, phone interviews were carried out with the parents of the children in order to obtain general information about them, as well as their health status and any physical or mental conditions that their children may be facing.

– Knowledge (K) & Practice (P) about general health and healthy habits for school children.

For K&P assessment, the widely adopted Bloom’s cutoff points are following: 80–100% (good K&P), 60–79% (moderate K&P), and less than 60% (poor K&P) [26,27,28]. In this study, we used the Median of the scores and a modified Bloom’s cutoff value with the subcategories of ‘Poor’ and ‘Fair’ scores grouped under the category ‘limited K&P’ about health and subcategories of ‘Good’ and ‘Excellent’ scores grouped under the category of ‘adequate K&P’ about health. These cutoff values were also based on previously published K&P studies [29,30,31,32]. Computed scores were graded into categories and subcategories, as shown in Table 1.

### 2.4. Statistical Analysis

Data were analyzed using SPSS version 25.

Univariate analysis was enrolled and all the study variables were presented. Nominal data (qualitative) were represented as frequencies and proportions. As for the continuous variables, results were presented by frequencies mean, standard deviation, minimum and maximum values.

The knowledge/good practice score was computed by adding the score of the knowledge and practice questions. Correct answers were coded by “1” and incorrect answers were coded by “0”.

Knowledge/Practice score pre- and post-educational intervention was presented by frequencies mean, standard deviation, minimum and maximum values. The Knowledge/Practice score was categorized to show poor, fair, good, and excellent Knowledge and Practice about general health and healthy habits for school children and was represented as frequencies and proportions.

The Knowledge/Practice score was assessed pre- and post-educational intervention. The improvement in knowledge was tested between the pre-Knowledge/Practice score and the post-Knowledge/Practice score using Paired Samples *t*-test.

The improvement was represented in function of all the study variables. Tests used in the bivariate analysis were Chi-square test and Fisher exact test.

The Knowledge/Practice score (continuous) was represented in function of all the study variables. Tests used in the bivariate analysis were independent *t*-test and ANOVA test. The Knowledge/Practice score (categorical) was represented in function of all the study variables. Tests used in the bivariate analysis were Chi-square test and Fisher exact test.

A statistically significant association was set at 5% (*p*-value less than 0.05).

### 2.5. Ethical Information

Before we started our study, under the supervision of Order of Malta Lebanon, we obtained approval from the Ministries of Public Health (MoPh)/Education (approval number 3/10460 received on 25 October 2022). This study was conducted in accordance with Good Clinical Practice ICH Section three, and the principles laid down by the 18th World Medical Assembly (Helsinki, 1964) [33] and all applicable amendments. Responses were confidential and were only used for research purposes. Parents and their children were asked by the school administration to sign electronically an informed consent in Arabic if they agreed to participate voluntarily in our study. In the informed consent, a detailed explanation of the background, objectives, risks, and advantages of the study was provided.

## 3. Results

### 3.1. Students Sociodemographic Characteristics

Out of the 7100 students between the ages of 3 and 12 years who underwent the awareness campaign, 16.11% of them (1144 students) aged 5 (39.2%), 8 (30.6%), and 11 (30.2%) years old were assessed before and after the campaign (Table S2).

Of the 1144 students who performed the interventional tests, approximately 75% of them were enrolled in private schools. The female population was slightly higher than the male population (52.4% vs. 47.6%), and the greater part of the participants were Lebanese (96.7%), with only a small percentage being Syrian. It is noteworthy that the majority of the study subjects hailed from rural areas in Lebanon, particularly from the Bekaa, Baalbeck, and North regions (Table S2).

According to the assessment of student habits and mental health in our campaign, only a small minority are alcohol (4.5%) and tobacco (7.2%) consumers. However, almost half (42%) of students were exposed to passive smoking in their homes for at least 1 day per week (18.2% of them for the whole 7 days/week). The majority (72.9%) of students also stated that they did not experience insomnia as a result of their concerns, with only a small percentage of students reporting such worries. Furthermore, a sizable portion of students (16.6%) reported that their parents or guardians never followed up with them to ensure that they completed their tasks, as opposed to 57.3% of those who said their parents always did so (Table S3).

Importantly, almost a quarter of the students were bullied at least one day per month. Other information regarding the students’ physical health and difficulties is represented in Table S4.

Information related to the student’s parents is also reported in Table S5.

### 3.2. Knowledge and Practices of Healthy Lifestyles for School Children Aged 5, 8, and 11 Years, According to Pre- versus Post-Interventional Tests

#### 3.2.1. Knowledge and Practice Overall Scores of Healthy Lifestyles

**Figure 3:**
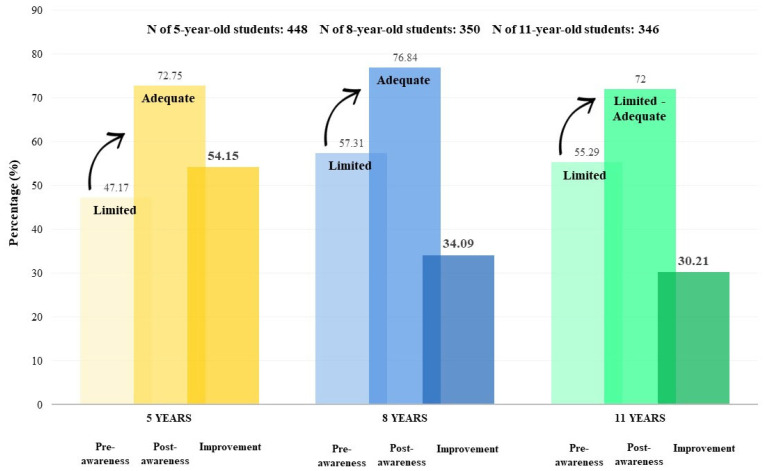
Mean percentage of correct answers of students aged 5, 8, and 11 years before and after the awareness campaign, along with their improvement (N = 1144).

By using the K&P scores to compare the average of correct answers in pre- vs. post-tests about health for each age group (5, 8, and 11 years old), students scored significantly higher in the post-test versus the pre-test (*p*-value < 0.001). Scores in the three age groups were particularly low (11.32 over 24, 17.46 over 32, and 18.80 over 34) in the pre-awareness campaign. Then, during the post-awareness campaign, the scores increased significantly in the three age groups, and the greatest improvement in the mean % of correct answers between the pre- and post-tests was observed in 5-year-old students (54.15% improvement), followed by 8-year-old students (34.09% improvement), and 11-year-old students (30.21% improvement). Table 2 and Figure 3 display these outcomes.

#### 3.2.2. Knowledge and Practice Scores of Healthy Lifestyles according to Categories and Subcategories

The impact of the awareness campaign on the K&P of 5-year, 8-year, and 11-year-old children was also represented in Figure 3, according to subcategories (poor, fair, good, and excellent awareness campaign) that were classified in Table 1. The results obtained during the pre-test showed that the 1144 participating students had an overall limited knowledge of healthy lifestyles in school children. Specifically, poor levels of K&P were recorded for all children (5, 8, and 11 years old) before the awareness campaign. The post-test results demonstrated an overall adequate K score with good levels of K&P for the three age groups.

**Figure 4:**
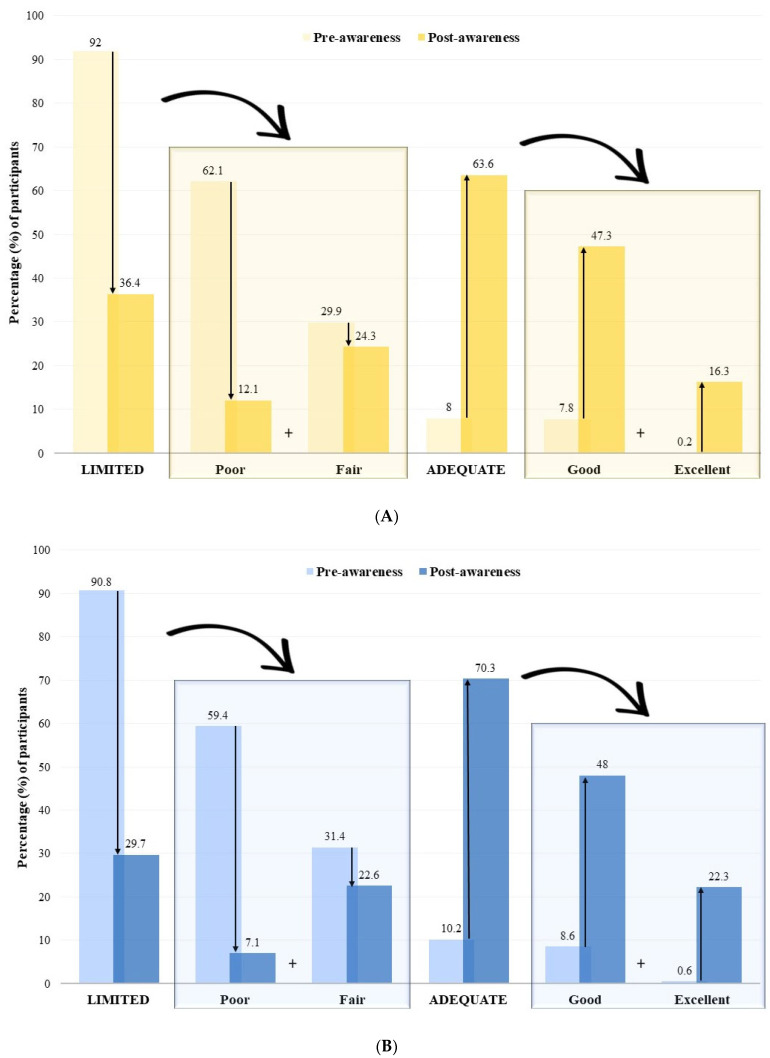

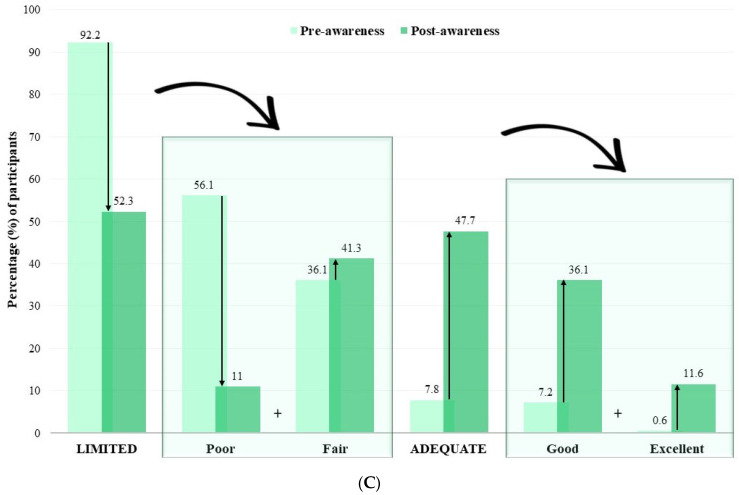
Distribution of (**A**) 5-year-old students (N = 448), (**B**) 8-year-old students (N = 350), and (**C**) 11-year-old students (N= 346) according to the knowledge and practice (K&P) score categories and subcategories.

- The 5-year-old students:

Before the awareness campaign, they had in the majority a limited health K&P (91.96%) (with 62.1% of students with poor K of healthy lifestyles and 29.9% of them with fair health K&P) and in the minority an adequate health K (8.03%) (with 7.8% of students with good K&P of healthy lifestyles and 0.2% of them with excellent K&P of healthy lifestyles) (Figure 4A). After the awareness campaign, while the proportion of 5-year-old students with limited K&P of healthy lifestyles decreased from 91.6% to 36.38% (with 12.1% of students with poor K&P of healthy lifestyles and 24.3% of them with fair K&P of healthy lifestyles), the proportion of students with adequate K&P of healthy lifestyles increased from 8.3% to 63.62% (with 47.3% of students with good K&P of healthy lifestyles and 16.3% of them with excellent K&P of healthy lifestyles) (Figure 4A).

- The 8-year-old students: a similar pattern was observed for the 8-year-old students, where the percentage of limited K&P of healthy lifestyles decreased from 90.86% to 29.71%, while adequate K&P of healthy lifestyles increased from 9.14% in the pre-test to 70.29% in the post-test (Figure 4B).
- The 11-year-old students: The majority of 11-year-old students had limited K&P of healthy lifestyles before the campaign, which decreased from 92.20% to 52.31% after the intervention. Adequate K&P of healthy lifestyles increased from 7.80% in the pre-test to 47.69% in the post-test (Figure 4C).

Overall, the percentage of students of the three ages with adequate K&P of healthy lifestyles increased significantly, while the percentage of students with limited K&P of healthy lifestyles decreased significantly, these data show that the awareness campaign was effective in improving the K&P of healthy lifestyles levels of the students in all three age groups.

#### 3.2.3. Knowledge Scores about Healthy Lifestyles by Variable

**Figure 5:**
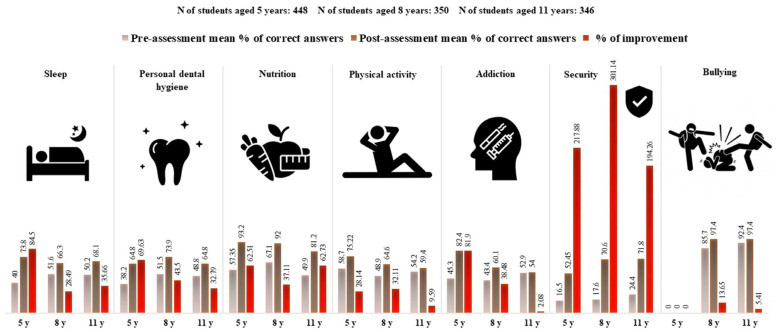
Knowledge and Practice (K&P) scores before and after the awareness campaign, along with improvement, across all addressed domains, in the 3 age groups (N = 1144).

The K&P score was also used to compare the correct answers for all variables obtained from the school children about healthy lifestyles for both pre- and post-tests. These parameters consist of the following: sleep, personal and dental hygiene, nutrition, physical activity, addiction, security measures, and bullying (Figure 5).

The results indicate that there was an overall improvement in the percentage of correct answers of all variables following the intervention. For instance, security-related questions showed the greatest increase in the mean percentage of correct responses for all three age groups. In contrast, questions where children aged 5 years displayed the least improvement were those related to physical activity, while for students aged 8 and 11, it was bullying. In addition, there was a slight improvement in addiction and physical exercise in students aged 11 years. This shows that the intervention led to an overall improvement in health K&P and habits among school children, with the majority of the three age groups being informed about security awareness and precautions, as evidenced by the significant increase in correct responses to security-related questions. Detailed responses of the students’ answers are provided in Table S1.

### 3.3. Factors Associated with the Improvement of Health Knowledge and Practice following the Intervention

A correlation analysis was conducted between the improvement of K&P toward health and habits following the awareness campaign and general characteristics (such as sociodemographic factors, comorbidities, habits, BMI, mental health of the children as well as the occupation, income, health K&P, level of education, marital status, smoking and comorbidities of the parents). The findings are presented in Table 3, Table 4, Table 5 and Table 6.

On a sociodemographic level, findings showed that there was no significant correlation between the improvement following the awareness campaign and the school type, school size, gender, age, and nationality of the children (*p* > 0.05). However, governorate was found to be a significant factor with the most improvement seen in both the South and the Beirut governorates while the least improvement was observed in the Baalbeck and the Bekaaa governorates (*p* < 0.001) (Table 3 and Figure S1).

Furthermore, our study found that comorbidities and developmental disorders and traits, such as dyslexia, dysphasia, ADHD, learning difficulties, and precocity did not have a significant impact on improvement except for dyspraxia, where students with this condition showed less progress compared to their peers who were not suffering from this condition. In terms of the students’ BMI, we observed a correlation between this parameter and improvement, with normal-weight students showing a more pronounced improvement than their overweight or underweight peers. Additionally, children who were overweight displayed a more noticeable gap in progress than their underweight counterparts (Table 4)

Regarding mental health, results revealed that students who were closely supervised by their parents or guardians better improved their health K&P score than those who were not (*p* = 0.021), and those who had worries that kept them up at night improved more than those who did not (*p* = 0.036). However, the students’ feelings of loneliness, sadness, stress, and peer bullying had no effect on their improvement score about health K&P (Table 5).

As for lifestyle habits, students who never smoked or those who were never exposed to passive smoking did not show an improvement in their health K&P score, when compared with smokers or those exposed to passive smoking, in opposition to alcohol usage where we had a significant correlation (Table 5). In terms of the influence of parental factors on their child’s improvement score about health K&P, we found that parents’ occupation, income, level of education, marital status, smoking, and comorbidities did not affect progress significantly (Table 6). Additionally, pregnancy and birth-related complications in the mother, such as low birth weight or prematurity of the child, did not significantly affect the improvement score about health K&P (Table 4).

## 4. Discussion

The findings from our research show that the school health awareness campaign was successful in raising children’s health awareness. This success highlights the potential of such initiatives as powerful tools for promoting better health knowledge and practices among children within educational settings. These results are consistent with numerous other studies that have shown how health awareness initiatives can increase children’s K&P of health-related subjects. For instance, according to Pulimeno et al., a health education intervention in schools effectively increased children’s K& P of healthy lifestyles [34]. Similarly, a study performed in Gampaha District, Sri Lanka by Radhik et al., concluded that school-based health education and awareness programs are valuable to uplift the level of awareness of students on diseases like dengue [35]. On a national scale, through a study conducted by the MUBS in Lebanon, researchers concluded that school health programs had the potential to make a significant contribution to the health and well-being of children in disadvantaged communities [16]. On the other hand, and in opposition to the findings of our research, other studies indicated that there may not always be a significant effect of school health awareness campaigns on children’s K&P about health. This may be explained by factors such as the duration, intensity, and design of the program, as well as the context in which it is implemented, which may play a role in determining the effectiveness of the intervention [36]. This is consistent with a Canadian study that looked at the impact of peer-led preventative oral health education for primary school-aged children and found that there was little change in oral health K&P [36]. Future studies must carefully take into account these elements to make sure that school health campaigns are planned and carried out in a manner that maximizes the influence they have on children’s health literacy.

### 4.1. Pre- and Post-Tests

Although questions in the pre- and post-intervention tests that took part in our study were tailored to the specific needs and characteristics of different age groups, the effectiveness of our campaign varied depending on the age of the children, with younger children potentially benefiting more from the intervention than older ones. This might be the result of several factors, such as the students’ stage of development, their degree of cognitive development, and their willingness to learn and experiment. This is in line with previous studies, where younger children were more responsive to health education interventions than their older peers. For instance, according to the Children’s Bureau of southern California, the period from infancy to age five is among the most important for development and learning [37].

Moreover, to investigate its impact on students’ dietary habits and nutrition, and through their project “*Colourful Means Healthy*”, a study conducted by Szczepańska et al. demonstrated the potential of school nutrition education in increasing healthy dietary habits knowledge among children [38]. Similarly, the findings of our study revealed a significant improvement in students’ nutrition knowledge following our intervention.

On top of that, our study showed a significant increase in security-related questions after the awareness campaign for all age groups. However, the mean percentage of correct answers did not reach the desired level, indicating room for improvement. Ongoing initiatives are essential to better prepare children for potential risks and threats. Regarding knowledge about bullying, there was only a minor improvement among 8- and 11-year-old children since levels of correct answers before the awareness campaign were already high. This suggests that the majority of kids in this age group are at least somewhat aware of the idea of bullying. However, it does not necessarily mean that they have a thorough awareness of the many types of bullying, nor does it mean that they will always be well-equipped to stop or deal with bullying when it occurs. In general, schools play a vital role in minimizing bullying and improving students’ knowledge of the issue. Olweus and colleagues’ study demonstrates that schools can lessen bullying and increase students’ understanding of the problem. They implemented a program in 42 Norwegian schools, and its impact on 2500 children were monitored. Only 20 months after the program ended, they discovered a decline in bullying for both girls and boys [39].

Furthermore, the average number of accurate responses for the other health variables assessed in our study increased following the intervention, suggesting that efforts to improve kids’ understanding of diverse health-related subjects can be helpful. The findings of a study conducted by Burke et al. are consistent with this; health programs that assessed 40 schools found that students’ knowledge, self-efficacy, and behaviors related to health had significantly improved over time [40]. However, in our study, the degree of change was encouraging but not as expected, indicating that more efforts are needed to increase children’s health literacy and that more effective intervention strategies may be required. By identifying these effective interventions, we can help kids gain the knowledge and abilities they need to make wise decisions about their health and well-being, which can have a long-term positive impact on their general health and quality of life.

### 4.2. Factors Associated with the Improvement of Health Knowledge and Practice (Figure S1)

Although school type, gender, age, and nationality of the children were not found to be significantly associated with the improvement of health K&P following the awareness campaign, yet, according to our research, the governate played a significant role in determining the success of the campaign. The rural area of Baalbek and Bekaa in Lebanon showed the least progress, whereas the urban area of the Beirut governate and the semi-urban area of the South governorate showed the most noticeable improvement. According to another survey, urban and semi-urban schools scored higher than rural schools in the following categories: “personal health and life skills,” “healthy school environment”, “health and nutrition services”, and “common disease control and prevention” [41]. Generally, studies on whether school health campaigns are more successful in rural vs. urban areas have produced conflicting findings. Possible explanations for this discrepancy include different lifestyles, values, and attitudes toward environmental conservation, emphasizing the importance of tailoring campaigns to the needs and circumstances of the target audience.

According to our research, there was no link between drug or tobacco use and children’s health improvement, but a strong connection with alcohol consumption. It is crucial to address youth alcohol use due to its harmful effects on health. Although smoking and drug use did not significantly correlate to health K&P progress, treating all forms of child substance misuse is important. The need to encourage good behaviors and guard against harm from substance addiction is crucial, as young children face increasing pressure to consume alcohol and use drugs [42]. Overall, combating child substance misuse is essential for their healthy and productive development.

Furthermore, the results of our study delved into how comorbidities, developmental disorders, and traits affect children’s development. While most of these conditions did not significantly impact progress post-campaign, dyspraxia had a negative effect. Research by Schoemaker et al. suggests that developmental disorders can affect areas beyond the core deficit domain, like mental health and quality of life [43]. Hence, it is crucial to consistently educate these children on healthy habits. Thus, children with developmental disorders may need specialized support and interventions for success and well-being.

In addition, the results of our study also provided insight into the effects of comorbidities, developmental disorders, and traits on children’s development. Despite the fact that the majority of these conditions did not appear to have a significant impact on improvement following the campaign, dyspraxia was found to have a negative effect. According to a study by Schoemaker et al., the effects of developmental disorders can be shown in other domains of development than the core deficit domain, such as mental health and health-related quality of life [43], for that, it is still crucial to consistently educate these children and incorporate information about healthy habits and topics related to health. Thus, children with developmental disorders and traits may require specialized support and interventions to help them reach success and well-being.

According to a recent study by epidemiologists at Brown University, children who were overweight or on the verge of becoming obese in their first two years of life scored lower on tests of perceptual reasoning and working memory than lean kids did when they were evaluated at ages five and eight, the study also suggested that children who are heavier may have lower IQ scores [44]. In another study carried out in western Ethiopia by Seyoum et al., it was found that underweight children had lower academic performance compared to those who were at a normal weight [45]. The results of the latter two pieces of research may support the finding of our study where students with a normal weight showed more improvement in their health knowledge following the intervention compared to their overweight or underweight peers. Interestingly, our study also found that overweight students displayed a larger gap in progress compared to their underweight counterparts. These findings suggest that being at a healthy weight may play a role in a child’s ability to benefit from health learning interventions.

Other findings from our study demonstrated a significant number of students who lacked parental academic supervision, impacting both their academic performance and overall well-being. Parental involvement is crucial for shaping attitudes towards education and fostering success, supported by the Modern University of Business and Science’s article “*Supporting School Health in Disadvantaged Rural Areas in Bekaa and Southern Lebanon*” on school health. Their research showed students with regularly involved parents performed better academically, leading to better study habits and reduced stress related to academic performance [16]. This aligns with the importance of parental involvement in educational achievement, as emphasized in a study on “The Importance of Parental Involvement in Education of Children” [46]. Our study supports these findings, highlighting the role of parental involvement in improving children’s health knowledge, where closely supervised students showed greater improvement in their K&P scores.

In addition, students who had worries that kept them up at night unpredictably improved more than those who had no significant worries. To our knowledge, no paper highlighted this association. One possible explanation for this finding is that children who reported having worries that kept them up at night may have been experiencing more pronounced symptoms of anxiety or depression, which could have motivated them to seek out more information and resources to manage their health.

In terms of the influence of parental factors on improvement, we found that parents’ occupation, income, health knowledge, level of education, marital status, smoking, and comorbidities did not affect progress significantly. This is in opposition with another study, which concluded that parents’ socioeconomic status affects student learning and academic achievements [47].

### 4.3. Recommendations

Given that health practices and behaviors are established during childhood, ensuring the well-being of school-aged children is crucial. For instance, it is essential to replicate this health campaign frequently in multiple schools to increase its influence on Lebanese communities. In order to create a strategy for future actions, it is also critical to discuss the results with the pertinent ministries since a significant proportion of students still fell under the ‘poor’ and ‘fair’ categories even after the campaign. This suggests that further efforts are needed to increase awareness and understanding among these students and to identify the most important characteristics of effective programs.

Furthermore, since parents play an important role in their children’s development, it may be important to encourage and support parents through a customized health awareness campaign with a pre- and post-test, this will encourage them to become more involved in their child’s education, particularly in monitoring their academic progress and overall wellbeing.

In addition, to effectively convey knowledge about health and foster critical thinking about risky behaviors among young people, it is important for educators to receive adequate training on how to boost students’ motivation toward healthy and sustainable lifestyles. This includes utilizing innovative and participatory methodologies that engage students and encourage them to think critically about the potential negative consequences of their actions.

### 4.4. Limitations

The study only considered schools that were close enough to OML medical centers and MMUs, which may not be an accurate representation of Lebanon’s general population. Therefore, results might not apply to all children of school age; rather, they may only apply to the population of kids who took part in the study.

Moreover, due to time restrictions prior to the campaign’s launch, nurses performing the intervention lacked the necessary training to transmit health knowledge and healthy behaviors and to ask questions in the pre- and post-tests in a child-friendly way. For that, the resulting changes in children’s health knowledge were not as great as anticipated, which may have had an impact on the success of the health awareness program. In addition, the health awareness program only lasted 40 to 45 min, which may not have been enough time to have a significant impact on the kids’ health knowledge and behaviors.

Furthermore, the data collection in our study was restricted to only one point in time following the intervention, which limits our capacity to track improvements in children’s health knowledge and healthy behaviors over time and the ability to assess the long-term effects of the intervention.

Finally, the study’s findings conflict with other studies on the same topic, indicating that the effectiveness of school health awareness campaigns may vary depending on various factors.

## 5. Conclusions

This school health awareness campaign boosted children’s understanding across diverse subjects including nutrition, bullying, physical activity, and dental hygiene, addressing areas where their knowledge was previously insufficient. It has not only effectively raised children’s health awareness but also highlighted a pivotal need for the primary educational institution to integrate more frequent student health promotions seamlessly into its regular teaching and learning procedures. By further embedding health-related initiatives within the educational framework, the school can continually foster a holistic approach to well-being, ensuring that students not only gain knowledge but also develop lifelong habits that contribute to their overall health and vitality.

## Figures and Tables

**Table 1:** Classification of the Knowledge and Practice (K&P) scores into categories and subcategories.

		Age Groups					
		5 Years		8 Years		11 Years	
Categories	Subcategories	Score/24	% of Correct Answers	Score/32	% of Correct Answers	Score/34	% of Correct Answers
Limited	Poor	≤12	≤50.00	≤19	≤59.37	≤19	≤55.88
	Fair	13–16	54.16–66.66	20–23	62.50–71.87	20–24	58.82–70.58
Adequate	Good	17–20	70.83–83.33	24–27	75.00–87.50	25–29	73.52–85.29
	Excellent	21–24	87.50–100	28–32	90.62–100	30–34	88.23–100

**Table 2:** Knowledge and Practice (K&P) scores before and after the awareness campaign (N = 1144).

Age Groups	Assessment Time	Score			% of Increase	Paired Differences		*p*-Value
		Mean Score ± SD	Over (Total Score)	Mean % of Correct Answers ± SD		Mean	SD	
5 Years Total = 448	Pre-awareness	11.32 ± 3.96	24	47.17 ± 16.50	54.15	−6.132	5.143	<0.001
	Post-awareness	17.46 ± 3.76		72.75 ± 15.67				
8 Years Total = 350	Pre-awareness	18.34 ± 4.04	32	57.31 ± 12.63	34.09	−6.251	5.131	<0.001
	Post-awareness	24.59 ± 3.52		76.84 ± 11.00				
11 Years Total = 346	Pre-awareness	18.80 ± 4.10	34	55.29 ± 12.06	30.21	−5.679	5.278	<0.001
	Post-awareness	24.48 ± 3.95		72.00 ± 11.62				

**Table 3:** Associations between sociodemographic factors and improvement of the health Knowledge and Practice (K&P) score following the awareness campaign.

Sociodemographic Factors		Improvement N (%)	
		No	Yes
Nationality Total = 1444	Lebanese Syrian Other	163 (14.7) 3 (8.6) 0 (0.0)	943 (85.3) 32 (91.4) 3 (100.0)—NS
Sex Total = 1444	Male Female	89 (16.3) 77 (12.9)	456 (83.7) 522 (87.1)—NS
Age Total = 1444	5 years 8 years 11 years	65 (14.5) 44 (12.6) 57 (16.5)	383 (85.5) 306 (87.4) 289 (83.5)—NS
Governorate Total = 1444	Beirut Mount Lebanon North/Akkar Beqaa/Baalbek Hermel South	6 (9.2) 54 (17.6) 38 (17.1) 48 (21.7) 20 (6.1)	59 (90.8) 253 (82.4) 184 (82.9) 173 (78.3) 309 (93.9)—*p* *
School type Total = 1444	Public Private	41 (13.9) 125 (14.7)	253 (86.1) 725 (85.3)—NS
OML community health facilities Total = 1444	Medical Mobile Units (MMUs) Medical centers	4 (5.3) 162 (15.2)	71 (94.7) 907 (84.8)—*p* *
	Barqa Khaldieh Zouk Kobeyat Room Ain El Remmaneh Seddiqine Kefraya	11 (4.9) 22 (18.0) 58 (18.4) 21 (17.5) 2 (5.6) 9 (21.4) 21 (14.2) 18 (29.0)	213 (95.1) 100 (82.0) 257 (81.6) 99 (82.5) 34 (94.4) 33 (78.6) 127 (85.8) 44 (71.0)—*p* *

*p* * = significant *p*-value. NS = non-significant *p*-value.

**Table 4:** Association between physical health and improvement of the health Knowledge and Practice (K&P) score following the awareness campaign.

Physical Health			Improvement N (%)	
			No	Yes
Body Mass Index (BMI)	5-year-old students Total = 448	Underweight Normal weight Overweight	8 (13.8) 41 (13.8) 16 (17.2)	50 (86.2) 256 (86.2) 77 (82.8)—NS
	8-year-old students Total = 350	Underweight Normal weight Overweight	12 (9.9) 9 (8.0) 23 (19.7)	109 (90.1) 103 (92.0) 94 (80.3)—*p* *
	11-year-old students Total = 346	Underweight Normal weight Overweight	14 (15.1) 11 (12.6) 32 (19.3)	79 (84.6) 76 (87.4) 134 (80.7)—NS
	All age categories Total = 1144	Underweight Normal weight Overweight	34 (12.5) 61 (12.3) 71 (18.9)	238 (87.5) 435 (87.7) 305 (81.1)—*p* *
Growth curve percentile	Weight Total = 1144	<5 5–25 26–50 51–75 76–95 >95	6 (17.6) 36 (18.2) 32 (15.8) 33 (11.5) 40 (13.8) 19 (14.1)	28 (82.4) 162 (81.8) 170 (84.2) 253 (88.5) 249 (86.2) 116 (85.9)—NS
	Height Total = 1144	<5 5–25 26–50 51–75 76–95 >95	8 (21.6) 24 (13.5) 23 (12.4) 35 (13.7) 48 (17.3) 28 (13.3)	29 (78.4) 154 (86.5) 162 (87.6) 220 (86.3) 230 (82.7) 183 (86.7)—NS
Pregnancy and birth condition	Normal Total = 533	No Yes Does not know	16 (14.8) 48 (11.3) 1 (50.0)	92 (85.2) 375 (88.7) 1 (50.0)—NS
	Low birth weight Total = 533	No Yes Does not know	60 (12.4) 4 (8.7) 1 (33.3)	424 (87.6) 42 (91.3) 2 (66.7)—NS
	Prematurity Total = 533	No Yes Does not know	61 (12.4) 3 (7.7) 1 (25.0)	429 (87.6) 36 (92.3) 3 (75.0)—NS
	Childbirth difficulties Total = 533	No Yes Does not know	60 (12.4) 4 (8.7) 1 (33.3)	424 (87.6) 42 (91.3) 2 (66.7)—NS
	Others Total = 533	No Yes Does not know	61 (12) 3 (13.6) 1 (50.0)	448 (88) 19 (86.4) 1 (50.0)—NS
Medical conditions currently or previously encountered	Early puberty Total = 524	No Yes Does not know	68 (13.3) 1 (11.1) 1 (25.0)	443 (86.7) 8 (88.9) 3 (75.0)—NS
	Late puberty Total = 524	No Yes Does not know	68 (13.3) 0 (0.0) 2 (33.3)	442 (86.7) 8 (100.0) 4 (66.7)—NS
	Diabetes Total = 524	No Yes Does not know	69 (13.3) 0 (0.0) 1 (20.0)	449 (86.7) 1 (100.0) 4 (80.0)—NS
	Hypothyroidism Total = 524	No Yes Does not know	69 (13.5) 1 (33.3) 0 (0.0)	442 (86.5) 2 (66.7) 10 (100.0)—NS
	Congenital heart disease Total = 524	No Yes Does not know	70 (13.6) 0 (0.0) 0 (0.0)	445 (86.4) 5 (100.0) 4 (100.0)—NS
	Dyslipidemia Total = 524	No Yes Does not know	69 (13.4) 1 (50.0) 0 (0.0)	445 (86.6) 1 (50.0) 8 (100.0)—NS
	Anemia Total = 524	No Yes Does not know	64 (13.3) 3 (12.0) 3 (16.7)	417 (86.7) 22 (88.0) 15 (83.3)—NS
	Asthma Total = 524	No Yes Does not know	69 (13.8) 1 (4.5) 0 (0.0)	430 (86.2) 21 (95.5) 3 (100.0)—NS
	Convulsions Total = 524	No Yes Does not know	70 (13.5) 0 (0.0) 0 (0.0)	449 (86.5) 2 (100.0) 3 (100.0)—NS
	Developmental dysplasia of the hip Total = 524	No Yes Does not know	70 (13.5) 0 (0.0) 0 (0.0)	448 (86.5) 1 (100.0) 5 (100.0)—NS
	Fractures Total = 524	No Yes Does not know	67 (13.5) 3 (11.1) 0 (0.0)	430 (86.5) 24 (88.9) 0 (0.0)—NS
	Cancer Total = 524	No Yes Does not know	70 (13.4) 0 (0.0) 0 (0.0)	452 (86.6) 1 (100.0) 1 (100.0)—NS
	Autoimmune disease(s) Total = 524	No Yes Does not know	68 (13.2) 0 (0.0) 2 (28.6)	446 (86.8) 3 (100.0) 5 (71.4)—NS
	Genetic disorder(s) Total = 524	No Yes Does not know	70 (13.6) 0 (0.0) 0 (0.0)	446 (86.4) 2 (100.0) 6 (100.0)—NS
	Chicken pox (Varicella) Total = 524	No Yes Does not know	67 (14.7) 3 (5.1) 0 (0.0)	389 (85.3) 56 (94.9) 9 (100.0)—NS
	German Measles (Rubella) Total = 524	No Yes Does not know	69 (13.6) 0 (0.0) 1 (7.7)	438 (86.4) 4 (100.0) 12 (92.3)—NS
	Measles Total = 524	No Yes Does not know	67 (13.5) 3 (17.6) 0 (0.0)	431 (86.5) 14 (82.4) 9 (100.0)—NS
	Mumps Total = 524	No Yes Does not know	67 (13.3) 2 (14.3) 1 (14.3)	436 (86.7) 12 (85.7) 6 (85.7)—NS
	Meningitis Total = 524	No Yes Does not know	70 (13.4) 0 (0.0) 0 (0.0)	451 (86.6) 0 (0.0) 3 (100.0)—NS
	Recurrent ear infections Total = 524	No Yes Does not know	56 (12.8) 14 (18.2) 0 (0.0)	383 (87.2) 63 (81.8) 7 (100.0)—NS
	Complicated urinary tract infections Total = 524	No Yes Does not know	63 (12.9) 6 (18.8) 1 (20.0)	424 (87.1) 26 (81.3) 4 (80.0)—NS
	Any chronic or recurring pain Total = 524	No Yes Does not know	66 (13) 4 (30.8) 0 (0.0)	440 (87) 9 (69.2) 5 (100.0)—NS
	Others Total = 524	No Yes Does not know	63 (13.1) 7 (17.1) 0 (0.0)	419 (86.9) 34 (82.9) 1 (100.0)—NS
Child difficulties	Learning difficulties Total = 509	No Yes Does not know	62 (14.3) 12 (16.4) 0 (0.0)	371 (85.7) 61 (83.6) 3 (100.0)—NS
	Dysphagia Total = 509	No Yes Does not know	65 (14.1) 9 (19.1) 0 (0.0)	396 (85.9) 38 (80.9) 1 (100.0)—NS
	Dyslexia Total = 509	No Yes Does not know	68 (14.8) 6 (12.8) 0 (0.0)	392 (85.2) 41 (87.2) 2 (100.0)—NS
	Dyspraxia Total = 508	No Yes Does not know	69 (13.9) 5 (50.0) 0 (0.0)	428 (86.1) 5 (50.0) 1 (100.0)—*p* *
	Precocity Total = 509	No Yes Does not know	39 (15.2) 35 (14.2) 0 (0.0)	217 (84.8) 212 (85.8) 6 (100.0)—NS
	Attention-deficit/hyperactivity disorder (ADHD) Total = 509	No Yes Does not know	70 (15) 4 (13.8) 0 (0.0)	397 (85) 25 (86.2) 13 (100.0)—NS

*p* * = significant *p*-value. NS = non-significant *p*-value.

**Table 5:** Association between lifestyle habits and mental health, and improvement of the health Knowledge and Practice (K&P) score following the awareness campaign.

Lifestyle Habits		Improvement N (%)	
		No	Yes
Age of smoking initiation Total = 1144	Never <7 years 8–9 years 10–11 years	162 (14.7) 1 (4.2) 0 (0.0) 3 (27.3)	940 (85.3) 23 (95.8) 7 (100.0) 8 (72.7)—NS
Number of days people have smoked in the student’s presence during the past 7 days Total = 1144	0 1–2 3–4 5–6 7	103 (15.5) 10 (9.7) 13 (11.9) 6 (9.8) 34 (16.3)	560 (84.5) 93 (90.3) 96 (88.1) 55 (90.2) 174 (83.7)—NS
Number of days the student has used tobacco products during the past 30 days Total = 1144	0 1–2 3–5 6–9 10–19 20–29 30	158 (14.9) 2 (10.5) 1 (7.1) 0 (0.0) 2 (25.0) 1 (20.0) 2 (6.9)	904 (85.1) 17 (89.5) 13 (92.9) 7 (100.0) 6 (75.0) 4 (80.0) 27 (93.1)—NS
Number of days the student has had at least one alcoholic drink during the past 30 days Total = 1144	0 1–2 3–5 6–9 10–19 20–29 30	154 (14.1) 10 (23.3) 0 (0.0) 0 (0.0) 1 (100.0) 1 (100.0) 0 (0.0)	939 (85.9) 33 (76.7) 2 (100.0) 2 (100.0) 0 (0.0) 0 (0.0) 2 (100.0)—*p* *
Chance to try an illegal drug (even if the student did not try it) Total = 1144	No Yes	166 (14.5) 0 (0.0)	975 (85.5) 3 (100.0)—NS
Mental health			
Homework checking by parents or guardians during the past 30 days Total = 1144	Never Rarely Sometimes Most of the time Always	39 (20.5) 6 (20.7) 10 (9.6) 16 (9.7) 95 (14.5)	151 (79.5) 23 (79.3) 94 (90.4) 149 (90.3) 561 (85.5)—*p* *
Feeling that the parents or guardians understood their problems and worries during the past 30 days Total = 1144	Never Rarely Sometimes Most of the time Always	44 (16.5) 10 (17.5) 16 (13.6) 24 (12.2) 72 (14.2)	222 (83.5) 47 (82.5) 102 (86.4) 172 (87.8) 435 (85.8)—NS
Feeling lonely during the past 12 months Total = 1144	Never Rarely Sometimes Most of the time Always	139 (15.7) 16 (9.9) 8 (10.4) 1 (9.1) 2 (22.2)	746 (84.3) 146 (90.1) 69 (89.6) 10 (90.9) 7 (77.8)—NS
Feeling so worried about something that they could not sleep at night during the past 12 months Total = 1144	Never Rarely Sometimes Most of the time Always	137 (16.4) 15 (8.1) 11 (10.9) 1 (8.3) 2 (18.2)	697 (83.6) 171 (91.9) 90 (89.1) 11 (91.7) 9 (81.8)—*p* *
Being sad or stressed during the past 12 months Total = 1144	Never Rarely Sometimes Most of the time Always	126 (15.8) 27 (13.0) 11 (9.0) 2 (14.3) 0 (0.0)	669 (84.2) 180 (87.0) 111 (91.0) 12 (85.7) 6 (100.0)—NS
Being Teased in a mean way or called hurtful names during the past 30 days Total = 1144	Never Rarely Sometimes Most of the time Always	137 (15.9) 16 (11.9) 11 (8.7) 2 (10.5) 0 (0.0)	722 (84.1) 119 (88.1) 115 (91.3) 17 (89.5) 5 (100.0)—NS
Number of days the student was bullied during the past 30 days Total = 1144	0 1–2 3–5 6–9 10–19 20–29 30	140 (15.5) 18 (12.9) 3 (5.4) 3 (21.4) 2 (16.7) 0 (0.0) 0 (0.0)	763 (84.5) 122 (87.1) 53 (94.6) 11 (78.6) 10 (83.3) 10 (100.0) 9 (100.0)—NS

*p* * = significant *p*-value. NS = non-significant *p*-value.

**Table 6:** Association between parent-related information and improvement of the health Knowledge and Practice (K&P) score following the awareness campaign.

Parent-Related Information			Improvement N (%)	
			No	Yes
Contacted parent’s rating of their own medical knowledge Total = 524	Bad Medium Good		3 (20.0) 32 (10.4) 35 (17.5)	12 (80.0) 277 (89.6) 165 (82.5)—NS
Contacted parent’s will to obtain medical knowledge Total = 524	No Yes		9 (22.0) 61 (12.6)	32 (78.0) 422 (87.4)—NS
Family Income Total = 490	No income <100$ 100–300$ 300–600$ 600–900$ >900$		10 (23.8) 31 (14.6) 8 (13.6) 15 (10.9) 5 (13.5) 0 (0.0)	32 (76.2) 181 (85.4) 51 (86.4) 123 (89.1) 32 (86.5) 2 (100.0)—NS
Contacted parent’s occupation Total = 513	Housewife Student Unemployed Employed Self-employed Retired Health field Other		38 (15.0) 1 (25.0) 19 (12.6) 4 (6.6) 1 (5.9) 3 (33.3) 2 (25.0) 1 (10.0)	215 (85.0) 3 (75.0) 132 (87.4) 57 (93.4) 16 (94.1) 6 (66.7) 6 (75.0) 9 (90.0)—NS
The other parent’s occupation Total = 465	Housewife Student Unemployed Employed Self-employed Retired Health field Other		10 (11.1) 3 (21.4) 25 (15.0) 11 (9.2) 9 (29.0) 2 (11.8) 2 (40.0) 3 (13.6)	80 (88.9) 11 (78.6) 142 (85.0) 108 (90.8) 22 (71.0) 15 (88.2) 3 (60.0) 19 (86.4)—NS
Contacted parent’s level of education Total = 524	No education Primary Complementary Secondary Undergraduate University graduate		0 (0.0) 7 (10.9) 20 (17.1) 19 (16.7) 7 (10.0) 17 (11.5)	11 (100.0) 57 (89.1) 97 (82.9) 95 (83.3) 63 (90.0) 131 (88.5)—NS
The other parent’s level of education Total = 504	No education Primary Complementary Secondary Undergraduate University graduate		0 (0.0) 8 (9.5) 17 (12.4) 23 (17.4) 6 (14.6) 12 (12.9)	17 (100.0) 76 (90.5) 120 (87.6) 109 (82.6) 35 (85.4) 81 (87.1)—NS
Contacted parent’s marital status Total = 490	Single Married Divorced Windowed		6 (23.1) 62 (13.9) 1 (7.1) 0 (0.0)	20 (76.9) 385 (86.1) 13 (92.9) 3 (100.0)—NS
The other parent’s marital status Total = 470	Single Married Divorced Windowed		3 (11.5) 60 (13.8) 1 (50.0) 1 (16.7)	23 (88.5) 376 (86.2) 1 (50.0) 5 (83.3)—NS
Contacted parent’s smoking status Total = 490	Never Former Occasional Current		37 (14.6) 7 (29.2) 5 (7.8) 20 (13.4)	216 (85.4) 17 (70.8) 59 (92.2) 129 (86.6)—NS
The other parent’s smoking status Total = 470	Never Former Occasional Current		30 (14.4) 5 (31.3) 6 (14.0) 24 (11.9)	179 (85.6) 11 (68.8) 37 (86.0) 178 (88.1)—NS
Medical conditions in the contacted parent Total = 490	Diabetes	No Yes Does not know	65 (13.9) 3 (14.3) 1 (50.0)	402 (86.1) 18 (85.7) 1 (50.0)—NS
	Heart disease	No Yes Does not know	64 (13.6) 3 (20.0) 2 (66.7)	408 (86.4) 12 (80.0) 1 (33.3)—*p* *
	Hypertension	No Yes Does not know	60 (13.4) 8 (21.1) 1 (33.3)	389 (86.6) 30 (78.9) 2 (66.7)—NS
	Dyslipidemia	No Yes Does not know	62 (13.8) 6 (15.4) 1 (33.3)	386 (86.2) 33 (84.6) 2 (66.7)—NS
	Previous stroke or thromboembolism	No Yes Does not know	68 (14.0) 1 (25.0) 0 (0.0)	417 (86.0) 3 (75.0) 1 (100.0)—NS
	Psychiatric disease(s)	No Yes Does not know	67 (14.1) 2 (15.4) 0 (0.0)	409 (85.9) 11 (84.6) 1 (100.0)—NS
	Cancer	No Yes	69 (14.1) 0 (0.0)	419 (85.9) 2 (100.0)—NS
	Osteoporosis	No Yes Does not know	67 (13.9) 1 (25.0) 1 (20.0)	414 (86.1) 3 (75.0) 4 (80.0)—NS
	Asthma	No Yes Does not know	68 (14.2) 1 (9.1) 0 (0.0)	410 (85.8) 10 (90.9) 1 (100.0)—NS
	Chronic disease	No Yes	63 (14.9) 6 (9.0)	360 (85.1) 61 (91.0)—NS
Medical conditions in the contacted parent Total = 470	Diabetes	No Yes Does not know	59 (13.5) 6 (18.8) 0 (0.0)	377 (86.5) 26 (81.3) 2 (100.0)—NS
	Heart disease	No Yes Does not know	63 (14.0) 1 (6.3) 1 (33.3)	388 (86.0) 15 (93.8) 2 (66.7)—NS
	Hypertension	No Yes Does not know	59 (14.2) 5 (9.6) 1 (33.3)	356 (85.8) 47 (90.4) 2 (66.7)—NS
	Dyslipidemia	No Yes Does not know	57 (13.4) 7 (16.7) 1 (33.3)	368 (86.6) 35 (83.3) 2 (66.7)—NS
	Previous stroke or thromboembolism	No Yes Does not know	64 (13.8) 1 (14.3) 0 (0.0)	399 (86.2) 6 (85.7) 0 (0.0)—NS
	Psychiatric disease(s)	No Yes Does not know	65 (14.1) 0 (0.0) 0 (0.0)	396 (85.9) 9 (100.0) 0 (0.0)—NS
	Cancer	No Yes	65 (13.9) 0 (0.0)	403 (86.1) 2 (100.0)—NS
	Osteoporosis	No Yes Does not know	63 (13.7) 0 (0.0) 2 (40.0)	396 (86.3) 6 (100.0) 3 (60.0)—NS
	Asthma	No Yes Does not know	64 (14) 1 (11.1) 0 (0.0)	394 (86) 8 (88.9) 3 (100.0)—NS
	Chronic disease	No Yes	61 (14.5) 4 (8.2)	360 (85.5) 45 (91.8)—NS

*p* * = significant *p*-value. NS = non-significant *p*-value.

## Data Availability

The data presented in this study are available on request from the corresponding author and OML. The data are not publicly available due to their containing information that could compromise the privacy of research participants.

## Supplementary Materials

The following supporting information can be downloaded at: https://www.mdpi.com/article/10.3390/children11020213/s1, Table S1. Detailed responses of students to the Knowledge and Practice Questionnaire (N = 1144). Table S2. Sociodemographic characteristics of participants. Table S3. Participants’ lifestyle habits and mental health. Table S4. Participants’ physical health and difficulties. Table S5. Parent-related information. Figure S1. Factors affecting the improvement of the students’ Knowledge and Practice (K & P) scores following the awareness campaign.
