# Supplementary material for: School-Based Pre- and Post-Intervention Tests Assessing Knowledge about Healthy Lifestyles: A National School Health Awareness Campaign on Children Aged between 3 and 12 Years Old
Source: Children (Basel). 2024 Feb 7;11(2):213. doi: 10.3390/children11020213 (PMC10887629; doi:10.3390/children11020213)
Supplement: Supplementary file 1 [file children-11-00213-s001.zip › children-2827270-supplementary.pdf]

children

Article

School-Based Pre & Post-Intervention Tests Assessing Knowledge about Healthy Lifestyles: A National School Health Awareness Campaign on Children Aged between 3 and 12 Years Old

Supplementary Materials

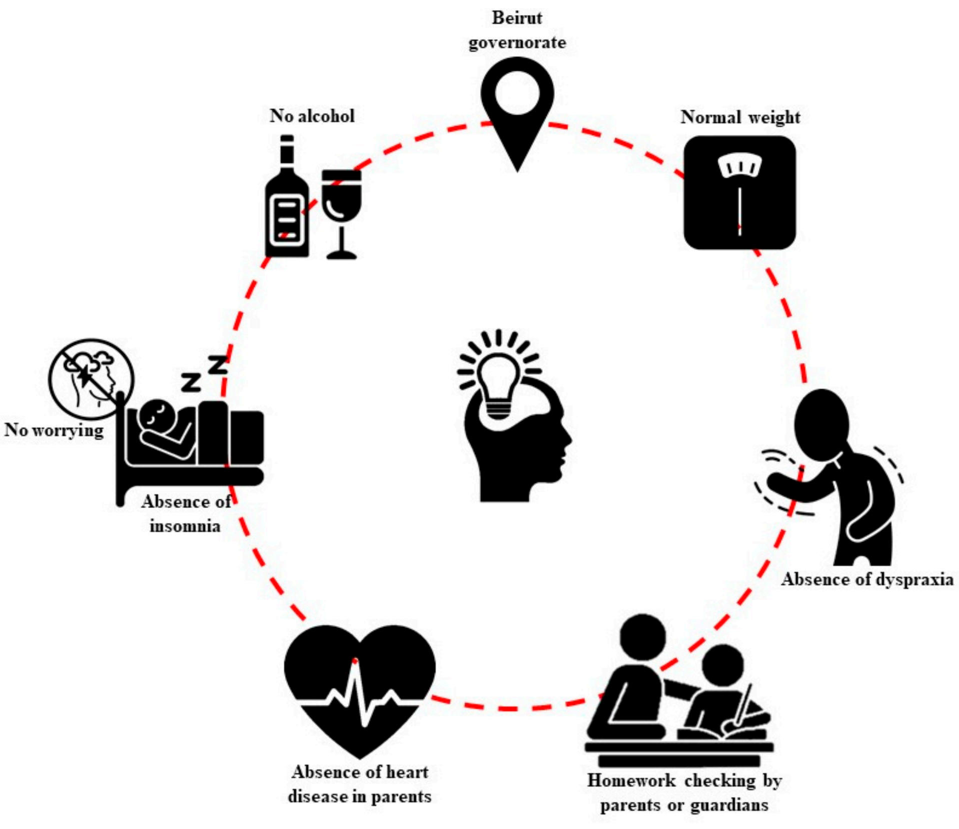

Figure S1. Factors affecting the improvement of the students' Knowledge and Practice (K & P) scores following the awareness campaign.

Table S1. Detailed responses of students to the Knowledge and Practice Questionnaire (N = 1144)

		Students aged 5 YEARS Total = 448 Answers		Students aged 8 YEARS Total = 350 Answers		Students aged 11 YEARS Total = 346 Answers	
**Questions**	**Options**	**Pre-assessment N (%)**	**Post-assessment N (%)**	**Pre-assessment N (%)**	**Post-assessment N (%)**	**Pre-assessment N (%)**	**Post-assessment N (%)**
**Sleeping help me to**	**Wrong**	80 (17.9)	20 (4.5)				
	**I don't know**	88 (19.7)	12 (2.7)				
	**Correct**	280 (62.5)	416 (92.9)				
**When you sleep well at night, what happens?**	**Wrong**			31 (8.9)	11 (3.2)		
	**I don't know**			32 (9.2)	1 (0.3)		
	**Correct**			287 (82)	338 (96.6)		
**At your age, how many hours should you sleep per night?**	**Wrong**	275 (61.4)	192 (42.9)	210 (60)	214 (61.2)	128 (37)	63 (18.3)
	**I don't know**	95 (21.3)	11 (2.5)	66 (18.9)	10 (2.9)	20 (5.8)	0 (0)
	**Correct**	78 (17.5)	245 (54.7)	74 (21.2)	126 (36)	198 (57.3)	283 (81.8)
**What habit(s) is good for sleeping well**	**Wrong**					210 (60.7)	195 (56.4)
	**Correct**					136 (39.4)	151 (43.7)
**When i sleep deeply**	**Wrong**					159 (46)	73 (21.1)
	**Correct**					187 (54.1)	273 (79)
**I have to take a shower**	**Wrong**	207 (46.3)	78 (17.5)	109 (31.2)	39 (11.2)	80 (23.2)	49 (14.2)
	**I don't know**	38 (8.5)	5 (1.2)	16 (4.6)	4 (1.2)	3 (0.9)	1 (0.3)
	**Correct**	203 (45.4)	365 (81.5)	225 (64.3)	307 (87.8)	263 (76.1)	296 (85.6)
**Choose the correct answer**	**Wrong**	203 (45.4)	184 (41.1)	106 (30.3)	58 (16.6)	188 (54.4)	143 (41.4)
	**I don't know**	68 (15.2)	11 (2.5)	13 (3.8)	1 (0.3)	3 (0.9)	1 (0.3)
	**Correct**	177 (39.6)	253 (56.5)	231 (66)	291 (83.2)	155 (44.8)	202 (58.4)
**I have to go to the dentist at least**	**Wrong**	195 (43.6)	147 (32.9)	188 (53.8)	158 (45.2)	220 (63.6)	162 (46.9)
	**I don't know**	120 (26.8)	48 (10.8)	77 (22)	14 (4)	37 (10.7)	9 (2.7)
	**Correct**	133 (29.7)	253 (56.5)	85 (24.3)	178 (50.9)	89 (25.8)	175 (50.6)
**What do you prefer to eat most of the time?**	**Junk Food**	138 (30.9)	46 (10.3)	88 (25.2)	18 (5.2)		
	**Healthy Food**	310 (69.2)	402 (89.8)	262 (74.9)	332 (94.9)		
**To me, healthy nutrition means that per day**	**Wrong**	203 (45.4)	105 (23.5)	136 (38.9)	38 (10.9)	94 (27.2)	29 (8.4)
	**I don't know**	41 (9.2)	2 (0.5)	6 (1.6)	0 (0.0)	6 (1.8)	1 (0.3)
	**Correct**	204 (45.6)	341 (76.2)	208 (59.5)	312 (89.2)	246 (71.1)	316 (91.4)
**Which fats are not considered good for our health?**	**Wrong**					247 (71.4)	98 (28.4)
	**I don't know**					0 (0)	2 (0.6)
	**Correct**					99 (28.7)	246 (71.1)
**What activities do you mostly practice**	**Other**	185 (41.3)	111 (24.8)	134 (38.3)	87 (24.9)	125 (36.2)	91 (26.4)
	**Sports**	263 (58.8)	337 (75.3)	216 (61.8)	263 (75.2)	221 (63.9)	255 (73.7)

**doing during the day?**							
**At my age, how many hours a day should I devote to sports?**	**Wrong**			191 (54.6)	156 (44.6)	180 (52.1)	188 (54.4)
	**I don't know**			33 (9.5)	5 (1.5)	12 (3.5)	2 (0.6)
	**Correct**			126 (36)	189 (54)	154 (44.6)	156 (45.1)
**How long MAXIMUM is it healthy at my age to spend in front of the screen (TV, computer, tablet, game console, phone…)?**	**Wrong**	245 (54.7)	79 (17.7)	307 (87.8)	256 (73.2)	281 (81.3)	284 (82.1)
	**Correct**	203 (45.4)	369 (82.4)	43 (12.3)	94 (26.9)	65 (18.8)	62 (18)
**Is smoking a cigarette bad for your health?**	**No it's not bad**			18 (5.2)	6 (1.8)	14 (4.1)	16 (4.7)
	**Maybe no it's not bad**			11 (3.2)	0 (0)	4 (1.2)	4 (1.2)
	**Maybe yes it's bad**			60 (17.2)	17 (4.9)	27 (7.9)	14 (4.1)
	**Yes it's bad**			261 (74.6)	327 (93.5)	301 (87)	312 (90.2)
**Do you know the number of the red cross (If yes, add the number in "others")?**	**No**	447 (99.8)	277 (61.9)	324 (92.6)	78 (22.3)	258 (74.6)	35 (10.2)
	**Yes**	1 (0.3)	171 (38.2)	26 (7.5)	272 (77.8)	88 (25.5)	311 (89.9)
**When you get in the car with your parents, do you put on the seat belt?**	**Never**	138 (30.9)	44 (9.9)	79 (22.6)	24 (6.9)	80 (23.2)	31 (9)
	**Rarely**	32 (7.2)	12 (2.7)	54 (15.5)	11 (3.2)	59 (17.1)	25 (7.3)
	**Sometimes**	94 (21)	60 (13.4)	91 (26)	66 (18.9)	97 (28.1)	68 (19.7)
	**Most of the time**	37 (8.3)	33 (7.4)	29 (8.3)	27 (7.8)	29 (8.4)	36 (10.5)
	**Always**	147 (32.9)	299 (66.8)	97 (27.8)	222 (63.5)	81 (23.5)	186 (53.8)
**Bullying happens when a student or a group of students says or does things bad and unpleasant to another student. Do you think bullying is:**	**Wrong**			15 (4.3)	0 (0)	15 (4.4)	8 (2.4)
	**I don't know**			35 (10)	9 (2.6)	11 (3.2)	1 (0.3)
	**Correct**			300 (85.8)	341 (97.5)	320 (92.5)	337 (97.4)

Table S2. Sociodemographic characteristics of participants

**Sociodemographic characteristics**				**Frequency**		**Percentage**
**Nationality**	Lebanese			1106		96.7
Total = 1144	Non-Lebanese			38		3.3
		Syrian	35		3.1	
		Other	3		0.2	
**Sex**	Male			545		47.6
Total = 1144	Female			599		52.4
**Age**	5 years			448		39.2
Total = 1144	8 years			350		30.6
	11 years			346		30.2
**Governorate**	Beirut			65		6.6
Total = 1144	Mount Lebanon			307		93.4
	North/Akkar			222		
	Beqaa/Baalbek Hermel			221		
	South			329		
**School type**	Public			294		25.7
Total = 1144	Private			850		74.3
**OML community**	Medical Mobile Units (MMUs)			75		25.7
**health facilities**	Medical centers			1069		74.3
Total = 1144		Barqa	224		19.6	
		Khaldieh	122		10.7	
		Zouk	315		27.5	
		Kobeyat	120		10.5	
		Room	36		3.1	
		Ain El Remmaneh	42		3.7	
		Seddiqine	148		12.9	
		Kefraya	62		5.4	

Table S3. Participants' lifestyle habits and mental health

**Lifestyle Habits**		Frequency	Percentage
**Age of smoking initiation**	Never	1102	96.3
Total = 1144	<7 years	24	2.1
	8-9 years	7	0.6
	10-11 years	11	1.0
**Number of days people have smoked in the**	0	663	58.0
**student's presence during the past 7 days**	1-2	103	9.0
Total = 1144	3-4	109	9.5
	5-6	61	5.3
	7	208	18.2
**Number of days the student has used tobacco**	0	1062	92.8
**products during the past 30 days**	1-2	19	1.7
Total = 1144	3-5	14	1.2
	6-9	7	0.6
	10-19	8	0.8
	20-29	5	0.4
	30	29	2.5
**Number of days the student has had at least one**	0	1093	95.5
**alcoholic drink during the past 30 days**	1-2	43	3.8
Total = 1144	3-5	2	0.2
	6-9	2	0.2
	10-19	1	0.1
	20-29	1	0.1
	30	2	0.2
**Chance to try an illegal drug (even if the student**	No	1141	99.7
**did not try it)**	Yes	3	0.3
Total = 1144			
**Mental health assessment**			
**Homework checking by parents or guardians**	Never	190	16.6
**during the past 30 days**	Rarely	29	2.5
Total = 1144	Sometimes	104	9.1
	Most of the time	165	14.4
	Always	656	57.3
**Feeling that the parents or guardians understood**	Never	266	23.3
**their problems and worries during the past 30 days**	Rarely	57	5.0
Total = 1144	Sometimes	118	10.3
	Most of the time	196	17.1
	Always	507	44.3
**Feeling lonely during the past 12 months**	Never	885	77.4
Total = 1144	Rarely	162	

	Sometimes	77	14.2
	Most of the time	11	6.7
	Always	9	1.0
			0.8
**Feeling so worried about something that they could not sleep at night during the past 12 months**<br>Total = 1144	Never	834	72.9
	Rarely	186	16.3
	Sometimes	101	8.8
	Most of the time	12	1.0
	Always	11	1.0
**Being sad or stressed during the past 12 months**<br>Total = 1144	Never	795	69.5
	Rarely	207	18.1
	Sometimes	122	10.7
	Most of the time	14	1.2
	Always	6	0.5
**Being Teased in a mean way or called hurtful names during the past 30 days**<br>Total = 1144	Never	859	75.1
	Rarely	135	11.8
	Sometimes	126	11.0
	Most of the time	19	1.7
	Always	5	0.4
**Number of days the student has been bullied during the past 30 days**<br>Total = 1144	0	903	78.9
	1-2	140	12.2
	3-5	56	4.9
	6-9	14	1.2
	10-19	12	1.0
	20-29	10	0.9
	30	9	0.8

Table S4. Participants' physical health and difficulties

**Physical health and difficulties-related parameters**			Frequency	Percentage
**Body Mass Index (BMI)**	Of 5 years old students	Underweight	58	12.9
Total = 1144	Total = 448	Normal weight	297	66.3
		Overweight	93	20.8
	Of 8 years old students	Underweight	121	34.6
	Total = 350	Normal weight	112	32.0
		Overweight	117	33.4
	Of 11 years old students	Underweight	93	26.9
	Total = 346	Normal weight	87	25.1
		Overweight	166	48.0
**Growth curve percentile**	Weight	<5	34	3.0
Total = 1144		5-25	198	17.3
		26-50	202	17.7
		51-75	286	25.0
		76-95	289	25.3
		>95	135	11.8
	Height	<5	37	3.2
		5-25	178	15.6
		26-50	185	16.2
		51-75	255	22.3
		76-95	278	24.3
		>95	211	18.4
**Pregnancy and birth**	Normal	No	108	20.3
**condition**		Yes	423	79.4
Total = 533		Doesn't know	2	0.4
	Low birth weight	No	484	90.8
		Yes	46	8.6
		Doesn't know	3	0.6
	Prematurity	No	490	91.9
		Yes	39	7.3
		Doesn't know	4	0.8
	Childbirth difficulties	No	484	90.8
		Yes	46	8.6
		Doesn't know	3	0.6
	Others	No	509	95.5
		Yes	22	4.1

		Doesn't know	2	0.4
**Medical conditions currently or previously encountered** Total = 524	Early puberty	No	511	97.5
		Yes	9	1.7
		Doesn't know	4	0.8
	Late puberty	No	510	97.3
		Yes	8	1.5
		Doesn't know	6	1.1
	Diabetes	No	518	98.9
		Yes	1	0.2
		Doesn't know	5	1.0
	Hypothyroidism	No	511	97.5
		Yes	3	0.6
		Doesn't know	10	1.9
	Congenital heart disease	No	515	98.3
		Yes	5	1.0
		Doesn't know	4	0.8
	Dyslipidemia	No	514	98.1
		Yes	2	0.4
		Doesn't know	8	1.5
	Anemia	No	481	91.8
		Yes	25	4.8
		Doesn't know	18	3.4
	Asthma	No	499	95.2
		Yes	22	4.2
		Doesn't know	3	0.6
	Convulsions	No	519	99.0
		Yes	2	0.4
		Doesn't know	3	0.6
	Developmental dysplasia of the hip	No	518	98.9
		Yes	1	0.2
		Doesn't know	5	1.0
	Fractures	No	497	94.8
		Yes	27	5.2
		Doesn't know	0	0
	Cancer	No	522	99.6
		Yes	1	0.2
		Doesn't know	1	0.2
	Autoimmune disease(s)	No	514	98.1
		Yes	3	0.6
		Doesn't know	7	1.3
	Genetic disorder(s)	No	516	98.5
		Yes	2	0.4

		Doesn't know	6	1.1
	Chicken pox (Varicella)	No	456	87.0
		Yes	59	11.3
		Doesn't know	9	1.7
	German Measles (Rubella)	No	507	96.8
		Yes	4	0.8
		Doesn't know	13	2.5
	Measles	No	498	95.0
		Yes	17	3.2
		Doesn't know	9	1.7
	Mumps	No	503	96.0
		Yes	14	2.7
		Doesn't know	7	1.3
	Meningitis	No	521	99.4
		Yes	0	0
		Doesn't know	3	0.6
	Recurrent ear infections	No	439	83.9
		Yes	77	14.7
		Doesn't know	7	1.3
	Complicated urinary tract infections	No	487	92.9
		Yes	32	6.1
		Doesn't know	5	1.0
	Any chronic or recurring pain	No	506	96.6
		Yes	13	2.5
		Doesn't know	5	1.0
	Others	No	482	92.0
		Yes	41	7.8
		Doesn't know	1	0.2
**Child difficulties**	Learning difficulties	No	433	85.1
Total = 509		Yes	73	14.3
		Doesn't know	3	0.6
	Dysphagia	No	461	90.6
		Yes	47	9.2
		Doesn't know	1	0.2
	Dyslexia	No	460	90.4
		Yes	47	9.2
		Doesn't know	2	0.4
	Dyspraxia	No	497	97.8
		Yes	10	2.0
		Doesn't know	1	0.2
	Precocity	No	256	50.3
		Yes	247	48.5

	Doesn't know	6	1.2
Attention-deficit/hyperactivity disorder (ADHD)	No	467	91.7
	Yes	29	5.7
	Doesn't know	13	2.6

Table S5. Parent-related information

**Parent-related information**		**Frequency**	**Percentage**
**Contacted parent's rating of their own medical knowledge** Total = 524	Bad	15	2.9
	Medium	309	59.0
	Good	200	38.2
**Contacted parent's will to obtain medical knowledge** Total = 524	No	41	7.8
	Yes	483	92.2
**Family Income** Total = 490	No income	42	8.6
	<100$	212	43.3
	100-300$	59	12.0
	300-600$	138	28.2
	600-900$	37	7.6
	>900$	2	0.4
**Contacted parent's occupation** Total = 513	Housewife	253	49.3
	Student	4	0.8
	Unemployed	17	3.3
	Employed	151	29.4
	Self-employed	61	11.9
	Retired	9	1.8
	Health field	8	1.6
	Other	10	1.9
**The other parent's occupation** Total = 465	Housewife	90	19.4
	Student	14	3.0
	Unemployed	31	6.7
	Employed	167	35.9
	Self-employed	119	25.6
	Retired	17	3.7
	Health field	5	1.1
	Other	22	4.7

**Contacted parent's level of education** Total = 524	No education		11	2.1
	Primary		64	12.2
	Complementary		117	22.3
	Secondary		114	21.8
	Undergraduate		70	13.4
	University graduate		148	28.2
**The other parent's level of education** Total = 504	No education		17	3.4
	Primary		84	16.7
	Complementary		137	27.2
	Secondary		132	26.2
	Undergraduate		41	8.1
	University graduate		93	18.5
**Contacted parent's marital status** Total = 490	Single		26	5.3
	Married		447	91.2
	Divorced		14	2.9
	Windowed		3	0.6
**The other parent's marital status** Total = 470	Single		26	5.5
	Married		436	92.8
	Divorced		2	0.4
	Windowed		6	1.3
**Contacted parent's smoking status** Total = 490	Never		253	51.6
	Former		24	4.9
	Occasional		64	13.1
	Current		149	30.4
**The other parent's smoking status** Total = 470	Never		209	44.5
	Former		16	3.4
	Occasional		43	9.1
	Current		202	43.0
**Medical conditions in the contacted parent** Total = 490	Diabetes	No	467	95.3
		Yes	21	4.3
		Doesn't know	2	0.4
	Heart disease	No	472	96.3
		Yes	15	3.1
		Doesn't know	3	0.6
	Hypertension	No	449	91.6
		Yes	38	7.8
		Doesn't know	3	0.6
	Dyslipidemia	No	448	91.4
		Yes	39	8.0
		Doesn't know	3	0.6

	Previous stroke or thromboembolism	No	485	99.0
		Yes	4	0.8
		Doesn't know	1	0.2
	Psychiatric disease(s)	No	476	97.1
		Yes	13	2.7
		Doesn't know	1	0.2
	Cancer	No	488	99.6
		Yes	2	0.4
	Osteoporosis	No	481	98.2
		Yes	4	0.8
		Doesn't know	5	1.0
	Asthma	No	478	97.6
		Yes	11	2.2
		Doesn't know	1	0.2
	Chronic disease	No	423	86.3
		Yes	67	13.7
**Medical conditions in the other parent** Total = 470	Diabetes	No	436	92.8
		Yes	32	6.8
		Doesn't know	2	0.4
	Heart disease	No	451	96
		Yes	16	3.4
		Doesn't know	3	0.6
	Hypertension	No	415	88.3
		Yes	52	11.1
		Doesn't know	3	0.6
	Dyslipidemia	No	425	90.4
		Yes	42	8.9
		Doesn't know	3	0.6
	Previous stroke or thromboembolism	No	463	98.5
		Yes	7	1.5
		Doesn't know	0	0.0
	Psychiatric disease(s)	No	461	98.1
		Yes	9	1.9
		Doesn't know	0	0.0
	Cancer	No	468	99.6
		Yes	2	0.4
	Osteoporosis	No	459	97.7
		Yes	6	1.3
		Doesn't know	5	1.1
	Asthma	No	458	97.4
		Yes	9	1.9
		Doesn't know	3	0.6

Chronic disease	No	421	89.6
	Yes	49	10.4
